# Potential Role for the Gut Microbiota in Modulating Host Circadian Rhythms and Metabolic Health

**DOI:** 10.3390/microorganisms7020041

**Published:** 2019-01-31

**Authors:** Shanthi G. Parkar, Andries Kalsbeek, James F. Cheeseman

**Affiliations:** 1 The New Zealand Institute for Plant & Food Research Limited, Private Bag 11600, Palmerston North 4442, New Zealand; 2 Department of Hypothalamic Integration Mechanisms, Netherlands Institute for Neuroscience, Royal Netherlands Academy of Arts and Sciences, Meibergdreef 47, 1105BA Amsterdam, The Netherlands; a.kalsbeek@nin.knaw.nl; 3 Department of Endocrinology and Metabolism, Amsterdam UMC, University of Amsterdam, Meibergdreef 9, 1105AZ Amsterdam, The Netherlands; 4 Department of Anaesthesiology, University of Auckland, Private Bag 92019, Auckland 1142, New Zealand; j.cheeseman@auckland.ac.nz

**Keywords:** sleep/wake rhythm, gut microbiome, plant food, prebiotics, clock genes, chronodisruption

## Abstract

This article reviews the current evidence associating gut microbiota with factors that impact host circadian-metabolic axis, such as light/dark cycles, sleep/wake cycles, diet, and eating patterns. We examine how gut bacteria possess their own daily rhythmicity in terms of composition, their localization to intestinal niches, and functions. We review evidence that gut bacteria modulate host rhythms via microbial metabolites such as butyrate, polyphenolic derivatives, vitamins, and amines. Lifestyle stressors such as altered sleep and eating patterns that may disturb the host circadian system also influence the gut microbiome. The consequent disruptions to microbiota-mediated functions such as decreased conjugation of bile acids or increased production of hydrogen sulfide and the resultant decreased production of butyrate, in turn affect substrate oxidation and energy regulation in the host. Thus, disturbances in microbiome rhythms may at least partially contribute to an increased risk of obesity and metabolic syndrome associated with insufficient sleep and circadian misalignment. Good sleep and a healthy diet appear to be essential for maintaining gut microbial balance. Manipulating daily rhythms of gut microbial abundance and activity may therefore hold promise for a chrononutrition-based approach to consolidate host circadian rhythms and metabolic homeorhesis.

## 1. Introduction

**Figure 1:**
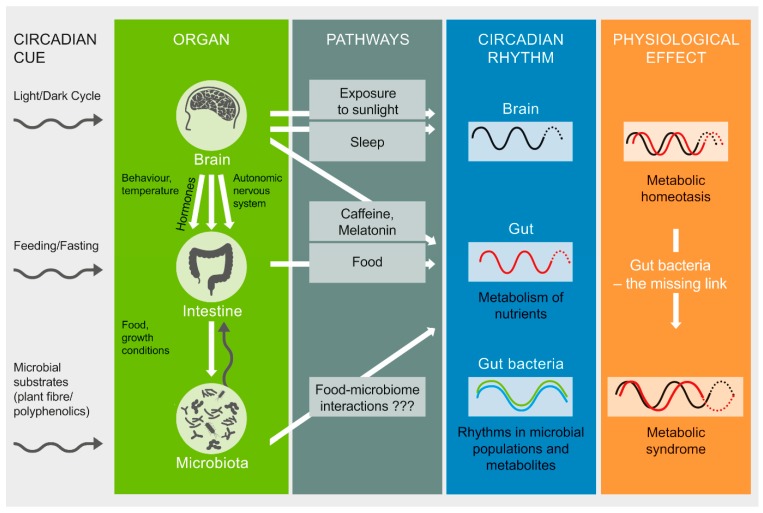
Circadian rhythm, gut microbiota, and metabolism. The light/dark cycle is the Zeitgeber for the central clock in the brain, while signals from the brain entrain peripheral clocks, e.g., in the intestine. Intestinal bacteria also show diurnal fluctuations in terms of their abundance and functions. Host controlled behaviors such as sleep, diet (food composition, timing of eating a meal), light exposure, and stimulants (e.g., caffeine) may potentially affect intestinal rhythms of metabolism. New evidence indicates that gut microbiota influences circadian rhythms, with consequent impact on the metabolic homeostasis of the host (Figure adapted from [1]).

Human metabolism is adapted to a circadian rhythm of ~24 h that is synchronized to the Earth’s 24 h light/dark cycle. This rhythm is orchestrated by the brain’s central clock in the hypothalamic suprachiasmatic nucleus , which in turn synchronizes peripheral clocks in the rest of the body (Figure 1). These clocks exist in almost every cell, and are comprised of a highly conserved set of core clock genes including period 1/2/3 (*Per1/2/3*), the brain and muscle aryl hydrocarbon receptor nuclear translocator-like 1 (*Bmal1*), cryptochrome 1/2 (*Cry1/2*), and the circadian locomotor output cycles kaput gene (*Clock*). This molecular clock mechanism plays an important role in regulating the rhythmic expression of clock-controlled genes, which in turn regulate the synthesis, storage, and expenditure of energy [1]. The light/dark cycle is the most potent Zeitgeber (an external or environmental cue that entrains the endogenous clock rhythms) for the central clock. Peripheral clocks may also be entrained by behavioral cues such as feeding and exercise [1,2]. 

The 24/7 lifestyles of shift work, early morning starts, delayed bedtimes, jet lag, and late night eating may cause circadian disruption, as our internal clocks may fail to keep pace with the conflicting information of the external light/dark cycle and our behavior in an optimal way. This misalignment of the rhythms that control our energy metabolism increases the risks of weight gain and metabolic syndrome, including type 2 diabetes mellitus [3,4]. Interestingly, an imbalance in our intestinal microbiota is also associated with obesity and diabetes [5,6,7,8].

The intestine affects our energy status by controlling physiological functions such as digestion and absorption of food, and gastric emptying—activities that are also regulated by clock genes [9]. The human intestine harbors a 10 trillion-cell microbial community that increases in abundance and diversity along the cephalocaudal axis with niche-specific variations in the spatial architecture of bacteria in the gut lumen and mucosal wall [10]. The five major bacterial phyla are Firmicutes, Bacteroidetes, Actinobacteria, Proteobacteria, and Verrucomicrobia, of which the former two constitute the vast majority, at relative abundances ranging up to 65% of total bacteria [11]. 

The single most abundant genus is *Bacteroides* (phylum Bacteroidetes), which thrives in the distal gut due to its metabolic flexibility, i.e., ability to break down a range of carbohydrates from dietary sources to host-derived mucins, thereby generating mainly acetate, succinate, and propionate [5,12]. Firmicutes, though poorer in carbohydrate breakdown enzymes, are enriched in genes involved in intracellular transport of nutrients including sugars [5]. This phylum, the most abundant genus of which is *Faecalibacterium* from the family Ruminococcaceae, generates most of the butyrate, which sustains the growth and integrity of colonic epithelial cells [11,12]. Actinobacteria, the third most abundant phylum, includes *Bifidobacterium*, which is considered a probiotic organism due to its versatility in terms of metabolizing dietary carbohydrates and ability to inhibit pathogens [11,12]. Of the low abundance phyla, Proteobacteria include commensal bacteria, many of which act as opportunistic pathogens [11,13,14]; while Verrucomicrobia, represented by the mucin-degrading *Akkermansia muciniphila,* is associated with improvement in metabolic health [15]. The human gut can comprise up to 1000 species, the number and proportions of which vary with a number of factors including mode of birth, age, gender, body mass, diet, health status, intake of medications especially antibiotics, activity, and travel [10,11,16]. This microbiome encodes about 5 million genes, which is ~100-fold greater than the human genome. The microbial metagenome includes the highly abundant machineries for carbohydrate degradation and utilization, synthesis of vitamins, detoxification of xenobiotics to the low abundance pathways for bile and sulfur metabolism. These functions are contained across multiple species that form a co-operative network to transform ecological niches to maximize survival of the microbial community [5,11,14].

The objective of this review is to explore the connections between gut microbiota and circadian rhythms by examining current literature using a set of questions to guide our understanding (Table 1). In the first part, we discuss current evidence on the gut microbiome-circadian rhythm interactions under different scenarios of circadian stress. In the second part, we examine gut microbiota-based mechanisms that may potentially protect hosts against the pathological manifestations of circadian disruption.

## 2. Factors Implicated in Gut Microbiome–Circadian Rhythm Interactions

In this section, we discuss external cues such as light/dark cycles and the consequent exposure to sunlight; and host-associated cues such as sleep and diet, on circadian–microbiome interactions with consequent effects on the host.

### 2.1. Light/Dark Cycles

Day/night rhythmicity has been seen in 20–83% of mice gut microbial taxa [17], much like the circadian oscillations seen in up to 50% of the mouse transcriptome [18]. Feeding restricted to the active dark phase resulted in a cyclical rhythm in the mouse gut microbiome, with Firmicutes peaking during feeding and decreasing during daytime fasting with a peak-to-trough ratio of 3:1. Bacteroidetes and Verrucomicrobia peaked during the daytime fasting period [17], potentially due to their greater ability to forage on intestinal mucin glycans [19]. Both dark phase and *ad libitum* feeding in mice, maintained dark phase peaks in *Bacteroidales* and light phase peaks in *Lactobacillales* and *Clostridiales*, along with expected increases in Firmicutes-associated functions such as detoxification of toxins, metabolism of vitamins, and environmental sensing, while the microbial rhythms were reversed by light-phase only feeding [20]. Such analysis of microbial abundance is generally expressed as relative abundance of different bacteria in a sample. Similar phyla-level rhythmicities were also observed in terms of relative abundance in male and female *ad libitum*-fed mice on 12/12 light/dark cycles, although the rhythmicities were more pronounced for female mice [21]. When comparing the data using the measure of *absolute* abundance of the bacterial groups, inferred from the product of relative abundance with the measured bacterial load, new patterns emerged. Diurnal oscillations were unchanged in Bacteroidetes, but lost in Firmicutes for both male and female mice, and the much less abundant Proteobacteria also showed light phase peaks in male mice [21]. These different mouse studies indicate that the peaks in Firmicutes during the dark phase are diet-driven, while blooms in Bacteroidetes, Verrucomicrobia, and Proteobacteria during the light phase were due to the cessation of feeding. One may thus conclude that periods of fasting are critical for ensuring the representation of bacteria that are otherwise outcompeted by the increase of Firmicutes after meal times, to break down dietary carbohydrates that reach the colon.

The diurnal rhythmicity of the microbial community is driven by the population dynamics of individual players and presence of their food. The more abundant Firmicutes thrive in response to the supply of dietary glycans and then their populations decrease when the food source is exhausted. The host intestinal mucosa is the major source of glycans during the rest phase. Bacteroidetes then dominate as they feed upon the host glycans using their wider repertoire of glycases, and penetrate deeper into host gut mucosal barriers [20,21]. Benefits to the host accrue in terms of energy and other growth factors such as vitamins, synthesized indigenously by the commensals. Consistent with this display of mutualism, *ad libitum* mice in normal light/dark cycles eat mainly in the dark phase, and this behavior resulted in an increase in the dark phase in microbial pathways related to energy metabolism, DNA repair, and cell growth. Furthermore, light phase resting led to an increase in microbial pathways related to chemotaxis and motility required for the mucus-adherent bacteria to reach closer to the intestinal wall [20,22].

Rhythmicity in bacterial adherence to the gut wall was influenced by multiple host factors. The effect of host’s feeding patterns was evident when microbial rhythmicity was phase-reversed by restricting the feeding to the light-phase in wild-type mice [20]. An intact host rhythm was also inferred to be important when gut microbial rhythmicity was lost in *ad libitum*-fed *Per*1/2 deficient mice [20]. However, again light-phase feeding restored microbial rhythms, indicating that host-controlled feeding behaviors, such as time-restricted feeding or fasting, can restore the rhythmicity lost after an impairment of the host’s clock machinery. A host circadian-gene control of the microbial rhythmicity of Bacteroidales, Clostridiales, and Lactobacillales, all major taxonomical orders in terms of function and abundance, was also inferred by the significant changes in their abundance in *Bmal1*-knockout mice [21]. However, this was most likely a consequence of rhythm loss in (feeding) behavior. Furthermore, examining the role of gender in microbiome rhythmicity, Liang et al. observed that female mice show a more pronounced amplitude for Bacteroidetes rhythms indicating potential hormonal influences on microbiota structure. This gender bias was much more subtle after *Bmal1*-deletion, detectable only at lower taxa levels [21]. Gut microbiota was affected in mice with mutations in the clock genes *Per1/2* (loss of microbial rhythms) and *Clock^∆19^* (loss of microbial richness and diversity), indicating a central role for host clock-controlled mechanisms in regulating gut microbiota [20,23]. Host clock-driven immune response mechanisms may also be responsible for gating the microbial community. For instance, the higher sensitivity of mice on 12/12 light/dark cycles to infection with the pathogen *Salmonella enterica* serovar Typhimurium in the early rest phase at Zeitgeber time 4 (i.e., ZT4, which is 4 h after lights on) rather than ZT16 (i.e., 4 h after lights off) [24]. How reversed feeding/fasting cycles, and the consequent light-phase peaking of the much more abundant Firmicutes may potentially outcompete the proteobacterial pathogens such as salmonella, was not explored in this study. This may be an interesting study given that the acidic by-products of metabolism by Firmicutes groups such as lactobacilli are known to selectively inhibit the growth of enteropathogens such as *Salmonella* [12,25].

Circadian disruption for a period of four weeks based on a regime of constant light exposure was also studied in *ad libitum*-fed mice. It was found to increase *Ruminococcus torques* and decrease *Lactobacillus johnsonii,* organisms that may have roles in impairing and protecting the integrity of intestinal barrier, respectively [26]. In the same study, bacterial metatranscriptomics data further demonstrated an increase in genes involved in the synthesis and transportation of endotoxin lipopolysaccharides, and decrease in genes that confer immune benefits to the host [26]. The role of light exposure on gut microbiota was also studied by subjecting *ad libitum*-fed mice to constant darkness for two weeks [27]. According to the authors, constant darkness disrupted microbial rhythmicity in both small and large intestinal segments, but sampling was not performed according to circadian time and the figures indicate a free-running rhythm rather than disruption. The authors also reported a significant clostridial bloom in the foregut. However, in both the light/light and dark/dark study, feeding activity, i.e., the key driver of microbial rhythms was not monitored. Light/dark cycles may thus influence the diurnal rhythms in resident commensals, but most likely this is by changing the feeding rhythm of the host. 

Rhythms in microbiome composition and metabolic activity have also been demonstrated in a human study which factored in clock time, and the timing and caloric density of the meals. In this study with 28 volunteers (male:female, 1:1), faeces samples were collected during normal waking hours between 07:30 and 22:00 h [28]. Over the day, butyrate producers, *Lachnospira*, *Roseburia*, and *Eubacterium* spiked earlier in response to the substrates made available due to the intake of food and decreased as the energy source was depleted after microbial utilization. Other bacterial groups that emerged earlier in the day included primary feeders that survive by rapid but incomplete breakdown of the carbohydrate to generate acetate and lactate (such as *Eggerthella*), the bile-tolerant groups (such as *Oscillospira* and *Bilophila*, that thrive in the post-prandial bile-rich intestinal milieu), and the H_2_S producing *Desulfovibrio* (in response to the increased lactate, and the sulfur from the bile taurocholates) [28,29]. 

As the intake of food decreased throughout the day, there was a shift in the gut microbiome, with a corresponding decrease in microbial metabolic processes that utilize the carbon sources to generate short chain fatty acids (SCFAs). Overnight fasting increased the SCFA propionate as the microbiota re-structured its community to forage on host-derived glycans [19]. Although faecal samples were not collected over the night period, this study demonstrated the effect of the clock time on gut microbial balance in humans. 

### 2.2. Sunlight Exposure

Light/dark cycles are well-recognized to be the most important cue for entrainment of the central clock in all mammals, including humans [1]. 

Insufficient exposure to sunlight might also affect us in other ways (as explained below), which potentially involve our gut microbiota. Exposure to the sun’s UV rays is essential for dermal synthesis of 25-hydroxyvitamin D (25-OHD, vitamin D3) which then enters systemic circulation [30]. Low levels of circulating vitamin D3 have been associated with visceral adiposity, which in turn increases susceptibility to insulin resistance and inflammation [31]. Vitamin D3 was found to increase the protective ileal mucus, which serves as an anchor for many beneficial bacteria including *Akkermansia muciniphila*, and also protects against colonization (and consequently breakdown of intestinal barrier) by opportunistic pathogens, such as adherent-invasive *Escherichia coli* [32,33,34]. Diet and supplementation with vitamin D3 for eight weeks was found to improve the gut microbial balance and richness in the upper gut (rather than the hindgut) in a small study of 16 healthy volunteers. There was a relative decrease in proteobacterial pathobionts such as *Pseudomonas* spp. and *Escherichia/Shigella* spp. [35]. Thus, circulating vitamin D3 levels can influence intestinal biogeography, and thereby microbial colonization. Further studies are required to investigate the effect of sunlight on the gut microbiome, and potential mediation of this effect by vitamin D.

### 2.3. Sleep

Insufficient sleep, duration, quality or timing, due to 24/7 schedules (e.g., jet lag, shift work) or choices (delayed bedtimes, lack of physical activity), and sleep disorders (sleep apnoea, narcolepsy), are associated with increased weight gain and impaired energy balance, glucose tolerance, and insulin sensitivity [3]. Sleep disturbance and the profound effect on circadian disruption contribute to these metabolic health outcomes [36].

Gut bacteria have been implicated in the regulation of sleep for nearly half a decade. In line with this, a study in rats showed that depletion of gut microbiota using antibiotics reduced slow wave sleep [37]. The effect of the broad-spectrum antibiotics, minocycline and amipicillin was studied in healthy male students (*n* = 19). Minocycline, but not ampicillin, reduced slow wave sleep for up to three consecutive nights after administration of the antibiotic, although there were no significant changes in sleep latency, nor wake after sleep onset [38]. Given the vast complexity of the gut microbiome in terms of composition, abundance, niche-specific, and consortia-specific resistance to antibiotics, it is difficult to correlate the role of specific bacterial groups with the regulation of sleep, let alone specific stages of sleep. Further studies are warranted to confirm microbiota-mediated effects on sleep given that minocycline is known to exert neuroprotective and anti-inflammatory effects on the central nervous system [39], which may potentially interact with the circadian system. 

An overgrowth of Gram-positive lactic acid bacteria results in the accumulation of d-lactate, which has been hypothesized to cause neurotoxic effects after crossing the blood–brain barrier [40]. Conversely, controlling the excessive accumulation of circulating d-lactate may therefore help to stabilize neurological symptoms. This hypothesis was tested in a human study where erythromycin (more effective against Gram positive bacteria) was given for six days to 22 patients (five male) with chronic fatigue syndrome. Faecal streptococci decreased significantly in seven participants, which correlated with improved sleep in terms of total sleep duration and sleep onset latency, and better subjective mood measures [40]. 

A preferential overgrowth of streptococci and other oropharyngeal, and colonic microflora has been observed in the gastrointestinal disorder, small intestinal bacterial overgrowth [41]. This microbial overgrowth has been implicated in the pathogenesis of restless legs syndrome, a sleep-related movement disorder, that typically worsens in the evening [42]. Indeed, bacteria or their cell components may be the cause of the inflammation seen with this disorder [16]. More study is warranted to investigate the connection between gut microbiome and the symptoms presented in sleep- and circadian-related disorders. 

How the gut microbiota is affected by sleep is of increasing interest given the integral role of microbiota in metabolic wellness. In one randomized crossover study with nine male volunteers, just two nights of partial sleep deprivation of 4 h (sleep opportunity 0245–0700 h), significantly decreased insulin sensitivity compared to normal sleep (22:30–07:00 h) during baseline, and after two days of sleep recovery. There were also significant changes to microbial populations that have been associated with a disturbed metabolism and even obesity (increased Coriobacteriaceae, Erysipelotrichaceae, and Firmicutes: Bacteroidetes ratio and decreased Tenericutes) [43]. However, a longer term study of two episodes of five nights of sleep restriction (sleep between 04:00–08:00 h), separated by five nights of 12 h sleep, with a final 12 h sleep recovery for two nights, did not show changes in the faecal microbial composition at baseline, during sleep restriction or after sleep recovery [44]. While both these studies were conducted in environmentally controlled laboratories, the latter study included both males and females (total *n* = 11), and collected faecal samples between 08:00–14:00 h to factor in for microbial rhythms. In the same study, the authors also conducted a 4 h-sleep restriction study in rats, subjected to seven days of forced activity in slowly rotating drums for 20 h. This sleep restriction did not alter any of the major phyla (compared to baseline), although it did increase a pro-inflammatory candidate phylum TM7-3a that was not restored after four days of sleep recovery. However, this regime of sleep restriction may not have caused sufficient disruption of the hosts’ circadian rhythms, which is emerging as a potential link to gut microbial rhythms. Chronic sleep disruption or sleep fragmentation such as that seen in sleep apnoea, also have an impact on body mass and insulin sensitivity. In a recent study in mice, four weeks of sleep fragmentation increased food intake and caused visceral adiposity and inflammation [45]. Overall, sleep fragmentation caused a shift in the major microbial phyla of the gut, lowering Actinobacteria by 50% and Bacteroidetes by 20%, and increasing Firmicutes by 20%, a profile characteristic of obese gut microbiota [10,16]. Significant changes were noted after two weeks of sleep fragmentation. There was a change in the relative abundance of two Firmicutes families, i.e., Lachnospiraceae (increase) and Lactobacillaceae (decrease). This microbial profile continued throughout the duration of sleep fragmentation protocol, although the bacterial abundance reverted to normal after two weeks of recovery from poor sleep. Circadian-sleep-gut microbiome connections were also inferred using metabolomic studies. Fifteen men volunteered for 24 h of sleep deprivation after a normal sleep/wake cycle with 8 h of sleep, under controlled conditions of light, sleep, meals, and posture. Acute sleep deprivation caused an increase in urinary metabolites, including those generated by gut bacteria. For example, acetate and 3-indoxyl sulfate, trimethylamine-N-oxide, p-cresol sulfate, and 3-hydroxyisobutyrate (generated by microbial breakdown of tryptophan, choline, tyrosine and valine respectively) [46]. 

Clearly, more studies are required to understand the connection between gut microbiota and modern lifestyles, which encompass inter-related factors i.e., sleep issues, circadian disturbances, stress, and poor dietary regimes that are well-recognized microbial manipulators. Indeed, dietary stressors such as alcohol or fat were found to exacerbate the gut microbiome disturbances caused by circadian disruption [23,47]. Light/dark shifts in mice on a diet containing alcohol increased the trend towards intestinal inflammation and polyposis, which at least partly may be contributed to by the increased Firmicutes: Bacteroidetes ratio that was driven by decreased *Allobaculum* and increased *Bacteroides* [48].

### 2.4. Jet Lag

Transmeridian or frequent multi-time-zone travel causes jet lag, a syndrome manifested by delayed physiological adaptation to rapid changes in time zone. The subjective effects of jet lag include poor mood, concentration, and bowel movement [49]. While there are multiple factors such as sleep, hormone rhythms, and dietary intake that are changed during jet lag, recent studies have demonstrated relationships between jet lag, gut microbial dysbiosis, and dysfunctional metabolic homeostasis. 

In one study, mice were subjected to four months of a simulated jet lag—multiple repeats of three days of an 8 h light cycle advance, followed by three days of the original light/dark cycle. Simulation of jet lag for four months did not significantly change the overall food intake, but did cause an increased weight gain and impaired glucose tolerance [20]. There was also a significant impact on gut microbiota and its functions, for example, the abundance and the rhythm of this abundance was suppressed for one of the major butyrate producing families, Ruminococcaceae. Broad-spectrum antibiotics given to jet lagged mice with gut dysbiosis rescued them from these metabolic changes, indicating a potential role for gut bacteria in inducing the metabolic pathologies of jet lag. That the effect of these dysbiotic microbial landscapes extended beyond the gut was proven when faeces from the time-shifted mice induced glucose intolerance in germ-free non-jet lagged mice [20]. It is thus possible that the loss of rhythms in microbially-mediated pathways associated with energy extraction and host maintenance contributed to this metabolic disruption. 

Short sleep duration on work days with sleeping in on free days, has resulted in the modern phenomenon of social jetlag [50]. In a cohort of 817 individuals, this social jetlag has been associated with predisposition to metabolic syndrome due to obesity, elevated glycated haemoglobin (a marker of prolonged elevation of blood glucose and consequent type 2 diabetes), and systemic inflammation [51]. While this study has not examined the impact on gut microbiota, changes in microbiome composition have been associated with these metabolic states. Gut microbial dysbiosis and the ensuing inflammation, and loss of intestinal integrity are postulated to be key mechanisms in causing metabolic disruption [52]. Dysbiotic patterns in diabetic individuals showa decreased Bacteroidetes, and changes in key Firmicutes such as lowered Ruminococcaceae, Lachnospiraceae, *Faecalibacterium prausnitzii*, and increased *Lactobacillus* indicates the putative connections between bacterial diversity (and functionality) and metabolic regulation of blood glucose [7,8]. 

### 2.5. Diet and Dietary Patterns

Circadian rhythms in the intestine are entrained both by the central clock and by food/eating patterns, and amongst others regulate the hosts’ intestinal cell transcription of the transport proteins required for uptake and absorption of the energy giving nutrients, glucose, amino acids, and triglycerides [9]. 

During undisturbed daily rhythm, for most humans, consumption of food will coincide with the activity phase, which in turn is synchronized with the light phase of the light/dark cycle, with consequent effects on the rhythms of circulating metabolites [53,54]. In a pilot study where 10 healthy male volunteers were subjected to 40 h of constant wakefulness, with enforced posture, dim light and hourly isocaloric meals, about 15% of the systemic metabolites displayed a circadian periodicity despite the lack of typical feeding/fasting routines, light/dark or sleep/wake cycles. The metabolites that peaked around subjective lunch-time were mainly lipid breakdown products such as fatty acids in the plasma and protein break down products, i.e., amino acids in the saliva [54]. This study, much like what may happen in a long-haul flight followed by a switch to a different time zone, indicates the inherent persistence of the internal clocks and metabolic features. The gut microbiota was not investigated in this study; although microbial populations have been shown to display periodicity in other studies in mice and humans [17,20,28]. 

While the central clock in the brain’s suprachiasmatic nucleusis a light-entrainable oscillator, peripheral oscillators (other parts of the brain, liver, gut, muscle, etc.) may be entrained by food, temperature or other factors, and even uncoupled from the suprachiasmatic nucleus by, for instance, restricted feeding during the sleep phase [1,2]. Indeed, the feeding regime is a powerful entrainer of peripheral clocks in metabolic tissues such as liver and intestine [2]. 

The feeding regime affects diurnal oscillations not just in body clocks, but also in the resident gut microbiota. A high-fat diet was found to alter oscillations in the brain, liver, and adipose tissue of clock genes and clock-controlled genes [55]. A high-fat diet was also shown to induce changes in the composition of the microbiome, the transplantation of which was able to induce obesity in germ-free mice [16]. In another study, a diet rich in fat and sugar exacerbated the effects of circadian disruption on the gut microbiota, with a drastic reduction of microbial diversity and the Firmicutes/Bacteroidetes ratio, mainly by promoting different bacteria from the Firmicutes phylum, most significantly Ruminococcaceae, altogether representing a microbiome such as that seen in obesity [10,16,47]. This gut microbial profile has also been observed in teenagers who consumed high sugar and fat diets [56]. A 20% increase in Firmicutes with a corresponding decrease in *Bacteroides* was associated with an increased energy harvest of 150 kcal/day, and this may be attributed to the differences in their genomic machinery to utilize and assimilate the substrates that reach the colon [57]. An energy surplus of this quantum over a year might result in body weight gain of about 5 kg per year. In conclusion, altered light/dark or sleep/wake cycles will have a profound influence on feeding patterns and circadian disruption, as the food that reaches the bowel would arguably be the primary driver for the changes in microbial growth. Thus, more studies are required to examine microbiome profiles that are a function of the geophysical cycle as opposed to feeding rhythms. 

Host diet has been known to rapidly alter the resident gut microbial composition and diversity [10,58,59]. Two major components that resist small intestinal digestion and reach the large intestine to regulate the host gut microbiome are the plant food-derived fiber and polyphenols [60,61]. Plant-based fiber and polysaccharides promote a butyrate-rich environment by enhancing proficient carbohydrate degrading bacteria from families such as Lachnospiraceae (*Roseburia*)*,* Eubacteriaceae (*Eubacterium rectale*), and Ruminococcaceae (*Ruminococcus bromii*), as compared to fiber-poor, animal-based diets, that favor the more bile tolerant *Alistipes, Bacteroides,* and *Bilophila,* that are more capable of protein breakdown [58]. When *Per2::Luc* (luciferase reporter gene fused to *Per2* gene) knock-in mice fed *ad libitum* with low fiber-containing chow were then shifted to diets containing 5% of the rapidly fermentable cellobiose or the slowly fermentable cellulose, there were significant effects on entrainment of peripheral *Per2* rhythms. The cellobiose-fed mice showed significant phase advances of *Per2* rhythm in the liver and kidney within 1 day, which was further increased on day 2. Cellulose, which is recalcitrant to microbial degradation, caused significant *Per2* phase advances only at the end of day 2, due to the slower build-up of SCFAs. *Per2* phase advances were seen even in submandibular salivary glands, albeit significantly only at day 2 [62]. 

Diet rich in galactooligosaccharides, polydextrose, lactoferrin, and milk fat globule membrane, components that are known to enhance beneficial bacteria, when administered to 24-day-old rats, was found to reduce the impact of stressor-exposure on gut microbial structure and diversity, and consolidate the sleep/wake cycle. There was no major change in any of the high-abundance bacterial phyla, but there was significant decrease in Deferribacteres and this was strongly correlated with longer non-rapid eye movement sleep [63]. 

The concentration and composition of the polyphenols in our diet may also potentially influence host circadian rhythms, albeit in peripheral tissues, by modulating the growth of gut commensals, which in turn generate bioactive SCFA or polyphenolic metabolites. For example, the polyphenol quercetin, ubiquitously found in plant foods, has a differential effect on the growth of gut commensals, with minimum inhibitory concentrations (MIC) being much lower for *Ruminococcus gauvreauii*, compared to *Enterococcus caccae* (MIC *R. gauvreauii* < MIC *Bifidobacterium catenulatum* < MIC *E. caccae*) [64]. While the latter two organisms produce lactate, the end product metabolite of *Ruminococcus* is butyrate, which has been shown to phase-advance hepatic clocks in mice (detailed in the SCFA section later) [65]. A decrease in the populations of Firmicutes (which includes both enterococci and ruminococci) compared to Bacteroidetes, results in an increased propionate/butyrate ratio in their metabolites, which was also observed with quercetin, at concentrations representative of a moderate consumption of a plant-based diet in an ex vivo simulation of the colon [66]. Thus polyphenols through their effects on the abundance and activity of gut bacteria may potentially influence the host circadian machinery. Furthermore, some polyphenols undergo microbial transformation to form metabolites, which may induce circadian entrainment. One such example is secoisolaricylresinol diglucoside (SDG), of which flaxseed is the richest source. SDG is minimally absorbed, and reaches the large intestine. SDG was shown to be metabolized by Firmicutes such as *Eggerthella lenta* and *C. coccoides-E. rectale* cluster to enterolactone [67,68]. A single dose of enterolactone (10 mg/kg body weight) was shown to influence canonical clock genes in 3 h in the murine uterus, by increasing the expression of *Clock* and decreasing that of *Per3* [69]. 

Meal timing and the eating window are now emerging to be just as important as the composition of the diet in entraining microbiome rhythmicity. In a study by Zarrinpar et al. [17], the gut microbiome was studied in mice fed a high fat diet only between ZT13 to ZT21 (ZT12 = lights off) or *ad libitum*. Mice on *ad libitum* regular chow were included as controls. After eight weeks, only the *ad libitum* high fat fed mice were obese with glucose intolerance. Dark phase restricted feeding elicited at least some microbiota changes that were closer to the regular chow fed microbial profile, and reversed, albeit partly, the obesogenic microbial profile caused by *ad libitum* high fat feeding. Time-restricted feeding (TRF; ZT13–ZT21) restored the cyclical circadian rhythmicity and the relative abundance of *Lactobacillus* spp. in the animals on the high-fat diet, to resemblethe *Lactobacillus* profile of the regular chow mice. TRF also decreased the abundance of *Lactococcus* spp. compared to the *ad libitum* high fat diet, which showed increased *Lactococcus* spp. Both these species are presumed obesogenic. TRF also restored the abundance, but not the cyclical rhythms of Clostridia and Ruminococcaceae such as *Oscillibacter,* during the active phase comparable to that seen with regular chow [17]. Indeed, microbial *rhythms,* such as those generated by TRF, may have greater metabolic consequences for the host than microbial abundances. Similarly, in humans, the length of overnight fasting (which may be considered as a form of TRF) was found to be proportional to the faecal propionate, which is channeled towards gluconeogenesis in the liver [12,28]. 

## 3. Gut Microbiota as a Potential Pathway to Restore Circadian Rhythm and Metabolic Health

Gut bacteria are increasingly recognized as being a critical organ within the human body, essential for development and maintenance of intestinal integrity and barrier, and optimal energy harvest. They play an important role in controlling the host’s metabolism and immunity [70,71]. Thus it should not come as a surprise that gut microbiota and its metabolites may also potentially influence host circadian rhythms [65]. We discuss here the microbial metabolites that might influence body clocks with outcomes in body weight and metabolic homeostasis (outlined in Table 2 and Table 3).

### 3.1. Secondary Bile Acids

Bile metabolism is an example of host and gut microbial activities, that are fixed to certain daily time points, as they co-ordinate metabolic homeostasis [72,73]. The liver secretes bile acids to facilitate the esterification and absorption of dietary fats and lipid soluble vitamins. The enterohepatic recycling of bile has a clear daily rhythm. Studies in mice demonstrate that the serum bile acids peak at the beginning and end of the dark phase. While most of the bile acids are resorbed by the liver from the distal ileum, some flow into the colon and undergo microbial metabolism, resulting in a serum peak of the secondary bile acids at the beginning of the dark phase [74,75]. This colonic microbial biotransformation includes deconjugation by bile salt hydrolases (*bsh*), followed by dehydroxylase and dehydrogenase activity, attributed to bacteria mostly from Firmicutes (Lachnospiraceae, Clostridiaceae, Erysepelotrichaceae, Ruminococcaceae, *Lactobacillus*), Bacteroidetes (*Bacteroides*), and *Bifidobacterium* [72,73]. *Bsh-*transformed *E. coli* colonization in both germ-free as well as conventionalized mice has confirmed the role of microbial deconjugation and biotransformation of bile acids in regulating hepatic and ileal clock genes (*Per1/2*), and clock controlled genes that regulate metabolism of lipid (*PPARγ, Angtpl4*) and cholesterol (*Abcg5/8*) [76]. 

Bile acids also shape the gut microbial landscape by affecting the membrane integrity of bile-sensitive Gram negative bacteria, many of which are pathogenic or pro-inflammatory due to their lipopolysaccharide endotoxins [72], and Gram positive bacteria such as lactobacilli and bifidobacteria [77]. A high cholic acid diet in rats caused a decrease in bile-sensitive *Bacteroides*, with a concomitant increase in Firmicutes, especially *Blautia* (class Clostridia) and *Allobaculum* (Erysipelotrichi) [77]. Thus, stressors such as a high-fat diet which lead to an arrhythmia in bile metabolism may potentially affect the rhythms of the gut microbiome composition and function (such as *bsh*), which in turn impact the rhythms of host metabolism, in this case, of lipids [77]. 

The increased levels of circulating bile acids in type 2 diabetic mice [78], indicates a much lower functional abundance of *bsh* in diabetes compared to control, which was corroborated with a metadata analysis [73]. 

### 3.2. Hydrogen Sulfide

The dissimilatory sulfite reductase (*dsr*AB), a low relative abundance microbial gene, is critical for the cycling of the sulfated compounds, such as the bile components and sulfated-mucins that reach the colon [14]. This *dsrAB* is present predominantly in Deltaproteobacteria such as *Desulfovibrio, Desulfotobacter, Desulfobulbus,* and the taurine utilising *Bilophila wadsworthia*, causing a net increase in H_2_S in the distal colon [13]. An increase in these Proteobacteria is prognostic of microbial dysbiosis. For example, the sulfate-reducing *Desulfovibro piger* has been shown to preferentially use up microbial lactate and competitively inhibit butyrate producers such as *Eubacterium hallii* and *Anaerostipes caccae*, that also require lactate [29]. The built up H_2_S is a powerful inhibitor of microbial functions, such as production of butyrate, a major source of energy in the colon, but also inhibits host functions, such as mitochondrial cytochrome c oxidase involved in the oxidative phosphorylation, thereby decreasing the amount of energy available for cell survival [79]. H_2_S has been shown to phase-delay *Bmal1* expression in mice hepatic organoids [65]. An increased microbial H_2_S (along with decreased butyrate) was observed along with suppressed substrate oxidation and elevated systemic glucose in high-fat fed mice [65]. Sulfate and taurine conjugates of bile and fatty acids, including sulfocholic acid, oxocholic acid sulfate, taurocholic acid sulfate, and cyprinol sulfate were found to be increased in faecal extracts of diabetic mice, indicating increased populations of intestinal sulfate-reducing bacteria in the diabetic mice [78]. 

### 3.3. Short Chain Fatty Acids

Gut bacteria break down digestion-resistant carbohydrates that reach the bowel to produce organic acids such as lactate and short SCFA metabolites such as acetate, propionate, and butyrate. Acetate and lactate are further utilized by secondary feeders to generate propionate and butyrate [12]. The acids not utilized in the colon enter the systemic circulation for absorption by peripheral tissues. The 4-carbon butyrate (more than the 3-carbon propionate or the 2-carbon acetate) plays a role in regulating plasma glucose by multiple mechanisms including activation of receptors that signal the secretion of satiety hormones, stimulation of insulin secretion, and suppression of pancreatic glucagon [6,80]. However, since acetate can also drive metabolism towards an obesogenic phenotype, as shown in a recent study with high-fat fed rats, it is important to have a full complement of gut bacteria to drive the carbohydrate fermentation to completion and enable the realization of microbiome-mediated metabolic benefits to the host [10,81].

Treatment with either 5mM acetate or butyrate, was found to modulate the expression of the clock genes, *Per2* and *Bmal1* in hepanoids that had been phase-synchronized with serum shock. Butyrate caused a significant change at the end of 8 h (*Per2* decrease by 0.5 fold, and *Bmal1* increase by 4-fold) [65]. Hepatic *Bmal1* expression was suppressed and *Per2* (and *Cry1*) increased in the dark phase for high-fat mice with guts colonized with specific pathogen-free microbiota. This effect on hepatic expression of clock genes was microbiota-mediated, and not observed when the gut was devoid of microbiota. The ex vivo experiments along with the in vivo observation confirm that microbiome, and its metabolites, at least partially explain the impact of five weeks of a high-fat diet on peripheral circadian rhythms in mice [65]. The same study also examined the effect of circulating butyrate on peripheral clock genes in mice at two different time points of the day. An intraperitoneal injection of butyrate (1000 mg/kg body weight) at ZT14 as opposed to ZT2 for 5 days, was found to increase *Per2:Bmal1* ratio (significantly in the liver, and approaching significance in the brain’s mediobasal hypothalamus) [65]. This study clearly indicates the microbiota–circadian link, although a high amount of plant-based fiber needs to be consumed and metabolized to butyrate at one time point, to achieve the concentrations of butyrate used in this study. Application of a mixture of SCFAs and lactate, at a concentration that may be achieved in vivo (100 µM), did not cause significant changes to *Per2* rhythms in mouse embryonic fibroblasts. There was, however, a dose-dependent (10 µM to 1 mM range) decrease in the amplitude of the first *Per2* peak, with significant change at 1 mM [62]. The concentration or the proportion of the individual components of this acid mixture are not given; it is possible that one or more of these acids (e.g., butyrate) may not have achieved the critical concentration required to induce *Per2* entrainment. Irrespective, this effect is of significant interest given that trends of circadian entrainment emerge with diet and adjusting meal timings rather than pharmacological intervention. 

Butyrate also potentially exerts an epigenetic control on circadian rhythms due to its ability to inhibit NAD^+^-dependent histone deacetylases such as sirtuin-1 (SIRT1) [82,83]. SIRT1 promotes the acetylation of BMAL1 associating with the CLOCK protein in the CLOCK:BMAL1 dimers [84]. SIRT1 thus plays a critical role in maintaining circadian rhythms and energy homeostasis via fat and glucose metabolism pathways, by promoting hepatic gluconeogenesis and fatty acid oxidation with effects on insulin sensitivity [85]. A note of caution though is that while butyrate and CoA individually act as histone deacetylase inhibitors, butyryl CoA acts an activator indicating that the effects of multiple derivatives of metabolites must be considered for their effects on histone deacetylases [86]. 

### 3.4. Vitamins

Some B vitamins have been directly or indirectly linked with circadian rhythms and sleep patterns [89,90]. While vitamins are often diet-derived, about 40–65% of gut bacteria have the genetic capacity to synthesize vitamins that are not produced by the host [11,91]. This metabolic capacity, distributed across different phyla, is an example of how microbiota can affect metabolism and behavior of its larger multi-cellular hosts (Table 3). The biosynthesis of vitamin B12, a complex small molecule that is essential for the survival of both host and the microbiome, exemplifies the co-operative conundrum that the commensal community establish for their survival, irrespective of their value to the host. Thus Veillonellaceae and Alphaproteobacteria may share the metabolic burden of synthesis of B12, but are co-dependent on the *Bacteroides thetaiotamicron* that navigate through the mucosal barrier to bind the dietary corrinoids (vitamin B12 precursors) and make them accessible to the microbiota for B12 synthesis [92].

### 3.5. Biogenic Amines

Sleep deprivation was found to cause a profound effect on the plasma metabolome, with an increase in biogenic amines or their precursors [99], which may be linked to increases in Firmicutes observed in sleep deprivation [43,45]. Dietary tryptophan is converted to serotonin in the host, and some of this transformation is attributable to microbial pathways given that germ-free mice show a 2.8-fold decrease in circulating serotonin [100]. Firmicutes such as *Clostridum sporogenes* and *Ruminococcus gnavus,* have been shown to harbor the tryptophan decarboxylase gene, homologs of which have been detected in 9–17% of healthy human gut metagenome [87]. Spore-forming clostridia further stimulate enterochromaffin cells to convert the end product tryptamine to the neurotransmitter serotonin. More than 90% of the body’s serotonin is gut-derived, and plays a role in peripheral functions including intestinal motility and secretory activity [88]. The microbial colonization of the intestine is also implicated in regulating brain serotoninergic pathways [101]. This in turn may influence the hippocampal concentrations of serotonin, a neurochemical that shows diurnal variations, and is implicated in promoting sleep and regulating hypothalamic pathways of glucose homeostasis [102].

A serotonin-derived bioamine, melatonin is implicated in regulating sleep/wake functions when synthesized in the brain. Gut-derived melatonin has been suggested to play a role in regulating insulin sensitivity and mitochondrial oxidation [103]. New studies indicate bidirectional associations between representatives of gut bacteria and host melatonin concentrations [104,105]. Intake of VSL#3 probiotics were also shown to improve sleep and intestinal symptoms in irritable bowel syndrome, which was associated with an increase in melatonin in early morning saliva samples [104]. On the other hand, gut bacteria are also responsive to bioamines in terms of their behavior and responses. *In vitro* studies indicated that pure cultures of *Enterobacter aerogenes* increase their swarming motility in the presence of melatonin [105]. This is particularly interesting given that *E. aerogenes* are opportunistic pathogens and produce pro-inflammatory endotoxins, and their presence significantly correlates with obesity [16]. It is thus possible that microbiota interactions with sleep-inducing biogenic amines potentially influence the circadian-metabolic axis.

### 3.6. Probiotics

Gut microbial dysbiosis and enteric infections are common factors in irritable bowel syndrome, and disrupted sleep is often associated as a co-morbidity [106]. Treatment with VSL#3, a combination of probiotic bacteria including *Bifidobacterium* (*B. longum*, *B. infantis,* and *B. breve*); *Lactobacillus* (*L. acidophilus*, *L. casei, L. delbrueckii* ssp. *Bulgaricus,* and *L. plantarum*), and *Streptococcus salivarius* ssp. *thermophilus* was found to improve sleep and abdominal pain in irritable bowel syndrome [104]. Although probiotic bacteria are exogenously administered and are often transient in their gut colonization [107], it is promising that close representatives of endogenous gut microbiota are implicated in sleep as well as gut health. It indicates the plausibility of prebiotic strategies to enhance endogenous beneficial organisms and restore health.

**Figure 2:**
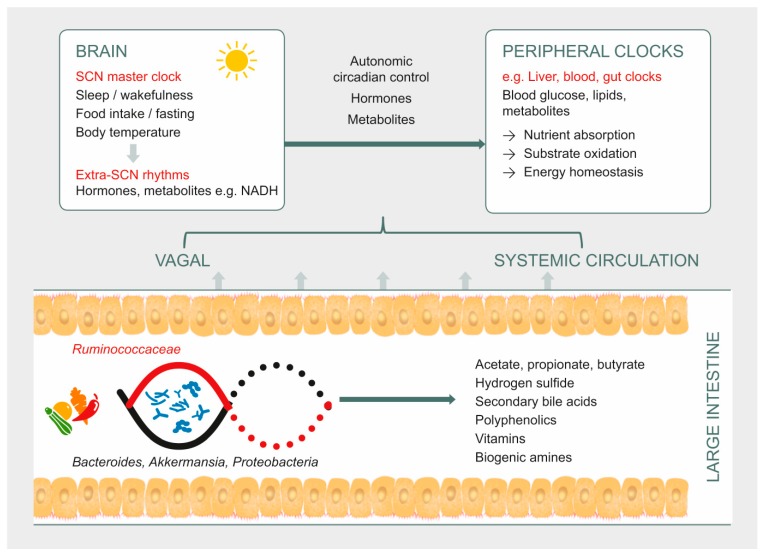
Potential mechanism of gut microbiota interactions in the circadian–metabolic axis. Host behavior, sleep, and diet control gut bacteria, which in turn show changes in terms of microbial composition and functional genome in a rhythmic manner. Circadian rhythms may be affected by microbial metabolites such as butyrate, secondary bile acids, and microbially synthesized vitamins, and potentially disrupted by hydrogen sulphide. The immediate effects on energy resources for colonic epithelial cells, and peripheral effects on substrate oxidation via systemic circulation potentially impact energy homeostasis.

In summary, host microbial rhythms may be considered a composite of peaks by the host-glycan foragers (primarily Bacteroidetes, but also Verrucomicrobia, and the opportunistic Enterobacteriaceae) anti-phase to thediet-driven Firmicutes peaks. Disruptions in sleep, diet, and eating patterns influence the diurnal dynamics of gut microbiome structure and activity. We propose a microbially-mediated mechanism of circadian disruption that involves alterations in the distinct time-of-day microbial signatures. If physiological adaptations to circadian disruption are not accompanied by gut microbial adaptation to time-of-day patterns, there may be metabolic consequences to the host in terms of the efficiency of energy extraction and utilization (Figure 2). For example, a decrease in bile metabolizing species and the consequent lowered *bsh,* may contribute to an increase in bile that in turn affects the rhythms in many bile-sensitive commensals while promoting sulfate reducers or pathobionts. The built up H_2_S caused by the bloom of sulfate reducers (increase in *dsrAB*) has been shown to strongly inhibit mitochondrial oxidation processes, and also phase-delay peripheral clocks. Sulfate-reducers also competitively inhibit the growth of butyrate producers, thus decreasing butyrate—a major energy source for colonic epithelium—potentially comprising epithelial integrity. The resulting dysbiosistriggers pro-inflammatory pathways leading to metabolic dysfunction—the hallmark of which is glucose intolerance. 

## 4. Conclusions

This review examines current evidence on the interdependency of host circadian systems and gut microbial ecology, and the consequences of this interaction for the host metabolism. There are many lifestyle features in the modern society that may contribute to the current epidemic of metabolic health problems such as obesity, type 2 diabetes, and metabolic syndrome. These features include, high fat/high sugar convenience foods and poor sleep due to modern-day stressors such as long working hours, multi-time-zone travel, and little exposure to daylight. All these lifestyle factors not only impact and disturb the circadian timing system, but also influence the make-up of our gut microbiome. The timing of eating a meal and the type of food may also affect circadian clocks, not just by host mediated cues but by virtue of the microbiota that it modulates. Both good sleep and a healthy diet, especially when time-of-day-appropriate, may thus be essential for maintaining metabolic homeostasis. 

The last five years have shown that the gut’s microbial residents also show a circadian rhythm in terms of their composition and function, and even their ability to invade and colonize the gut. Current evidence indicates an increase in total gut bacterial mass and Firmicutes, in response to the food ingested during the waking/eating phase, and an increase in Bacteroidetes, Proteobacteria and Verrucomicrobiaduring the sleeping/fasting phase. There is also evidence of diurnal variation in the microbial metabolites such as butyrate and H_2_S, which in turn affects the host’s circadian rhythm and metabolism. For example, pathobionts such as *Desulfovibrio* and the bile-tolerant *Bilophila* increase during the day and produce H_2_S. 

Manipulating the microbiome might therefore be a promising strategy to restore the host’s circadian rhythm and metabolic homeostasis. Plant foods are a rich source of fiber and polyphenols, the undigested components of which reach the colon to generate beneficial metabolites including SCFAs and polyphenolic bioactives, which in turn not only maintain colonic health and protect against pathogens, but also might help resynchronize circadian rhythms. For example, one may expect increases in Ruminococcaceae and Lachnospiraceae with a high fiber diet, and subsequent generation of butyrate to support the energy requirements of the intestinal cells during the day, and provide extra entrainment to the peripheral organs such as the liver and potentially brain. 

We also discussed the contributing pathways and gut microbiota-based mechanisms that may rescue the host from the pathological manifestations of circadian disruption. These include microbial generation of bioactive SCFAs, polyphenolic metabolites, vitamins, and bioamines. This is particularly valuable because diet offers natural therapeutic strategies to modulate our microbiota. We acknowledge that in isolation, none of the above factors solve the metabolic issues arising from chronic circadian disruption, but given that modern lifestyles are here to stay, dietary manipulations are probably something that consumers are willing to try. Because many components in food can alter gut microbial composition and functions, it is possible that manipulating the quality, quantity, or timing of food, may manipulate our gut microbiota and ultimately influence and mitigate some of the metabolic consequences of modern lifestyle-associated issues such as disrupted sleep and disorganized circadian rhythms.

## Figures and Tables

**Table 1:** Guiding queries used to examine gut microbiome–circadian interactions.

Query	Conditions	Examples of Evidences
How does circadian rhythm affect gut microbiota?	Light/dark cycles	Effect of diurnal variation and altered light/dark cycles on gut microbiome
	Sleep status	Gut microbiome in sleep deprivation/sleep fragmentation
	Diet	High fat diet, night eating
How does gut microbiota affect circadian rhythm?	Antibiotic-induced gut microbial alterations	Do antibiotics affect sleep or circadian-controlled metabolic states?
	Microbial dysbiosis due to intestinal disease	Chronic gut disorders with altered microbiota showing co-morbidities in sleep
	Microbial metabolites	Short chain fatty acids, Secondary bile acids
		Vitamins
		Biogenic amines
		Hydrogen sulfide

**Table 2:** Major microbiota-mediated mechanisms that influence host circadian and metabolic pathways *.

Microorganisms	Microbial Function	Interactions with Host Pathway
Firmicutes (Lachnospiraceae, Clostridiaceae, Erysepelotrichaceae, Ruminococcaceae, *Lactobacillus*), Bacteroidetes (*Bacteroides*), and *Bifidobacterium* [72,73].	Microbial bile salt hydrolases deconjugate bile deoxycholic acid and lithocholic acid [76].	Microbial bile salt hydrolase associated with modulation of canonical clock genes, genes related to lipid metabolism and immune homeostasis [76].
*Desulfovibrio, Desulfotobacter, Desulfobulbus, Bilophila wadsworthia* [13].	Act on sulfated compounds to generate hydrogen sulfide in the colon.	Hydrogen sulfide phase-delays hepatic *Bmal1* expression, which is also associated with suppressed substrate oxidation and elevated systemic glucose [65].
Lachnospiraceae (*Roseburia*)*,* Eubacteriaceae (*Eubacterium rectale*), and Ruminococcaceae (*Ruminococcus bromii, Faecalibacterium prausnitzii*) [11,12]	Break down dietary fiber to generate butyrate in the colon [58].	Butyrate is a key metabolic fuel for colonic epithelial cells [11,12]; regulates plasma glucose by multiple mechanisms including activation of receptors that signal the secretion of satiety hormones, stimulation of insulin secretion and suppression of pancreatic glucagon [6,80]; modulates canonical clock genes in peripheral tissue [65]; acts as a histone deacetylase inhibitor [82,83].
*Clostridum sporogenes* and *Ruminococcus gnavus* [87].	Generate precursors of biogenic amines such as serotonin [87].	Bioamines such as serotonin play a role in intestinal motility and secretory activity [88].

* Microorganisms related to synthesis of vitamins elaborated in Table 3.

**Table 3:** Microbially synthesized vitamins and their physiological effects through circadian-controlled mechanisms.

Vitamin	Physiological Effects Through Circadian Controlled Mechanisms	Examples of Microorganisms That Synthesize the Vitamin
**B1, thiamine**	Core body temperature, rat study [93]	*Streptococcus thermophilus* ST5, *Lactobacillus helveticus* R0052, *Bifidobacterium longum* R0175 [94]
**B2, riboflavin**	Affects metabolism by influencingcryptochrome 2 stability [95]	*Bacillus subtilis*, *Escherichia coli* [94]
**B3, niacin**	Lowered blood B3 associated with decreased duration of deep sleep in Parkinson’s disease [96]	*E. coli* [91]
**B9, folic acid**	Important for brain function by regulating brain clock genes [97]; lowered serum B9 associated with sleep disturbance [90]	*Bifidobacterium* spp., *Lactobacillus* spp. [94]
**B12, methylcobalamine or cyanocobalamine**	Lowered serum B12 associated with sleep disturbance [90]; also reverses hydrogen sulfide effects on substrate oxidation [98]	*Propionibacterium freudenreichii*; *Lactobacillus reuteri* [94]
